# Multivariate Spectral Analysis of Transabdominally Recorded Intrauterine Acoustic Signals (TRIAS) Across Gestation

**DOI:** 10.3390/s26134150

**Published:** 2026-07-01

**Authors:** Ryo Tamaki, Fuyuka Igarashi, Ryutaro Yamamoto, Hiroshi Asano, Akira Oku, Keiichiroh Akabane, Kiwamu Noshiro, Ami Hosokawa, Yoshihiro Saito, Hidemichi Watari, Takeshi Umazume

**Affiliations:** Department of Obstetrics, Hokkaido University Hospital, Kita–ku N15 W7, Sapporo 060-8638, Japan

**Keywords:** gestational age, non-invasive monitoring, principal component analysis (PCA), partial least squares regression (PLSR), spectral analysis, TRIAS

## Abstract

**Highlights:**

**What are the main findings?**

- Identification of a significant power spectral density (PSD) decrease in the 400–600 Hz range (specifically 478.5 Hz and 521.5 Hz) during the second trimester.
- Evidence that transabdominally recorded intrauterine acoustic signals (TRIAS) may reflect gestational progression, with maternal heart rate contributing as a major source of acoustic variability.

**What are the implications of the main findings?**

- The proposed multivariate analytical framework provides a foundational methodology for decoding complex, overlapping physiological signals toward non-invasive monitoring.
- These findings provide an exploratory baseline for integrated maternal–fetal monitoring, with potential future application to clinical and home-based settings.

**Abstract:**

This study proposes a multivariate analytical framework for transabdominally recorded intrauterine acoustic signals (TRIAS) and evaluates spectral changes across gestation. Using a digital stethoscope, 60 recordings were obtained from 44 pregnant women (≥14 weeks). Power spectral density (PSD) was calculated (30–2000 Hz) and analyzed using ordinary least squares (OLS) regression, principal component analysis (PCA), and partial least squares regression (PLSR). A consistent spectral peak was observed at 300–400 Hz. OLS revealed a significant decrease in PSD levels within 400–600 Hz (specifically 478.5 Hz and 521.5 Hz) during the second trimester (*t* ≈ −3.5), while changes were minimal in the third trimester. PCA identified maternal heart rate as the primary contributor to the first principal component, and PLSR showed that maternal cardiovascular dynamics and gestational progression were associated with distinct, largely independent components in the multivariate space. These results suggest that TRIAS may capture a composite maternal–fetal acoustic environment influenced by both maternal circulation and gestational uterine alterations. Specifically, the observed second-trimester spectral changes may reflect fluid dynamic or structural transitions—such as variations in amniotic fluid volume—and may serve as a baseline for future monitoring research.

## 1. Introduction

The development of fetal assessment methods remains an important challenge in perinatal care. As pregnancy progresses, the fetus undergoes rapid growth and organ maturation, while its ability to adapt to environmental changes and complications is still limited. Therefore, early detection of abnormalities and appropriate intervention are essential for improving the prognosis of both the mother and fetus [1]. Currently, ultrasonography and fetal heart rate monitoring (cardiotocography, CTG) are established as standard evaluation methods [1,2]. However, these techniques require regular hospital visits and are not suitable for long-term monitoring. In addition, adequate followup may be difficult in regions where access to health care is limited [3].

Changes in the intrauterine milieu are known to affect fetal development through multiple pathways, including blood flow, oxygen delivery, and neural development. If such changes could be monitored over time, they might enable earlier detection of abnormalities [4]. Biological sounds recorded on the maternal abdomen contain diverse acoustic components, including fetal heartbeat, fetal movement, umbilical cord blood flow, and amniotic fluid flow, as well as maternal sounds such as breathing and bowel activity [5,6,7]. The intrauterine acoustic environment to which the fetus is exposed may change with advancing gestational age [8]. To date, the evaluation of fetal behavior and physiological responses has often relied on acoustic measurements using Doppler and other methods, and the analytical frameworks capable of capturing the overall structure of multidimensional and continuous acoustic data remain limited.

In recent years, digital stethoscopes equipped with high-sensitivity microphones have become widely available, enabling non-invasive recording of biological sounds from the maternal abdomen through the abdominal wall. In this study, we hereby introduce the term “transabdominally recorded intrauterine acoustic signals (TRIAS)” to describe the composite acoustic environment—comprising fetal, uterine, and maternal components—captured via digital stethoscope on the maternal abdominal surface. Parga et al. recorded abdominal sounds from pregnant women using an electronic stethoscope and reported that their frequency characteristics varied with gestational age [5]. Riknagel et al. recorded uterine artery blood flow sounds using an electronic stethoscope and reported that the presence or absence of a dicrotic notch, a characteristic waveform feature, was significantly associated with reduced placental function [9]. The same group further reported an association between maternal vascular murmurs and fetuses with intrauterine growth restriction accompanied by abnormal umbilical artery Doppler waveforms [10]. Eiríksdóttir et al. developed an automated method for assessing the signal quality of maternal and fetal cardiovascular sounds recorded from the skin surface near the uterine artery and demonstrated its utility for clinical applications [11]. These passive acoustic monitoring methodologies, including conventional fetal phonocardiography [11,12], complement established obstetric modalities and offer significant advantages for low-cost, decentralized screening. More broadly, controlled acoustic and ultrasound-based technologies are increasingly recognized for their capacity to interact non-invasively with reproductive biomaterials over extended periods [13]. Within this evolving technological landscape, TRIAS may serve as a non-invasive spectral signature reflecting cumulative gestational changes and macro-environmental alterations in the uterine milieu.

However, most previous studies have relied on conventional analysis approaches, such as univariate comparisons at individual frequencies, and have not systematically explored or visualized multivariate patterns across the acoustic spectrum. In addition, a standardized analytical framework suitable for clinical application has not yet been established, and methods for the comprehensive evaluation of TRIAS are still evolving.

In this study, we applied multivariate analysis to high-dimensional, continuous intrauterine acoustic data recorded from the maternal abdominal wall and proposed a corresponding analytical framework with potential clinical application. TRIAS comprise multiple physiological sound sources, including fetal heartbeat, fetal movement, and umbilical cord blood flow, as well as maternal heartbeat, respiration, and intestinal peristalsis. As a result, TRIAS represent a multicomponent and nonstationary acoustic signal. Despite this complexity, to our knowledge, no studies have systematized these acoustic signals as clinical data or examined their relationship with gestational age in a multidimensional manner. To address this gap, we employed principal component analysis (PCA) and partial least squares regression (PLSR) to analyze the multicomponent structure of TRIAS. PCA is a widely used method for reducing high-dimensional data and visualizing the variance structure [14,15], while PLSR is a regression technique that maximizes covariance between explanatory variables and outcome variables and has been applied in biosignal analysis [16]. Moreover, analytical frameworks that integrate time-varying spectral information with multivariate analysis have proven effective for extracting pathology-related patterns from high-dimensional data in other fields, such as serum nuclear magnetic resonance (NMR) analysis [17,18,19]. In the present study, we extend this analytical concept to the investigation of TRIAS.

## 2. Materials and Methods

### 2.1. Participants

This observational study was conducted to characterize TRIAS during pregnancy. Pregnant women with singleton pregnancies who were receiving antenatal care at our institution were recruited for the study. Eligible participants were aged 18 years or older and were at ≥14 weeks of gestation at the time of recording. Exclusion criteria included a pre-pregnancy body mass index (BMI) ≥ 25 kg/m^2^, the presence of major pregnancy complications (including hypertensive disorders of pregnancy and gestational diabetes mellitus), fetal growth restriction, or any other medical condition that was considered by the attending physician to be unsuitable for study participation. Some participants underwent multiple acoustic recordings during the course of pregnancy. Therefore, the dataset includes repeated measurements obtained from the same individuals at different time points.

### 2.2. Acoustic Recording

TRIAS were recorded non-invasively using a digital stethoscope (3M^TM^ Littmann^®^ CORE Digital Stethoscope; 3M Company, Maplewood, MN, USA). During recording, participants were positioned in a supine position in a quiet environment to minimize ambient noise interference, and the stethoscope was positioned on the maternal abdominal wall at the midpoint between the umbilicus and the pubic symphysis. To maintain geometric consistency across variations in fetal position and uterine fundal height, this anatomical landmark was kept uniform for all participants. To minimize physiological and environmental artifacts, all recordings were conducted by two designated, well-trained operators who followed a standardized measurement protocol, including the application of consistent, light contact pressure to support inter-operator reproducibility. In addition, identical device settings were maintained across all recording sessions to limit device-induced spectral scaling artifacts. Participants were instructed to remain in a comfortable supine position, maintain quiet, controlled breathing, and refrain from speaking or moving during the 60-second continuous recording. Acoustic data segments contaminated by transient gross maternal movements or loud bowel sounds were inspected visually and auditorily and excluded prior to spectral analysis. This device was selected for its broad-frequency sensitivity (30–2000 Hz) and its high-fidelity digital output suitable for subsequent signal processing [5,9,10]. The acoustic data were captured at a sampling rate of 44.1 kHz with 16-bit quantization. Based on the effective frequency response of the device, the analysis frequency range was initially set to 30–2000 Hz. However, subsequent analyses were restricted to 30–1000 Hz. First, in vivo transmission models confirm that the maternal abdominal wall, myometrium, and amniotic fluid function as a physical acoustic low-pass filter; while transmission loss is minimal below 500 Hz, acoustic energy above this threshold undergoes severe exponential tissue attenuation, physically extinguishing or burying high-frequency intrauterine components beneath the system’s baseline noise floor [6,7]. Consequently, dynamic physiological signals from the gestational cavity are naturally confined below 1000 Hz [5], whereas surface data within the 1000–2000 Hz range represent a stable, non-dynamic background noise floor (Figure S1). Second, including this uninformative high-frequency noise floor in the multivariate pipeline would introduce stochastic variance, causing mathematical overfitting in the PCA and PLSR frameworks. Truncating the features at 1000 Hz was therefore required to isolate genuine relative physiological changes. Although the hardware-specific frequency response of the digital stethoscope influences the absolute baseline power density, the standardization of all recording parameters supports the interpretation that the observed variations across the mid-frequency bands reflect genuine relative physiological changes rather than instrumental artifacts.

### 2.3. Spectral Analysis

All acoustic data (WAV format) were analyzed using MATLAB (R2025a, MathWorks, Natick, MA, USA). For each 60-second recording, the power spectral density (PSD) was estimated using Welch’s method with a 512 ms Hamming window and 50% overlap, yielding a frequency resolution of approximately 1.95 Hz. Normalization was performed by subtracting the mean PSD across the analysis frequency range (30–2000 Hz) from the PSD at each frequency, correcting for differences in absolute sound intensity and enabling comparison of relative spectral shapes across recordings. The relationship between PSD level at each frequency and gestational age was evaluated using a linear regression model based on ordinary least squares (OLS). The rate of change in PSD level with respect to gestational age was quantified as the regression slope (β1, dB/week) for each frequency. Gestational age-related frequencies were identified by focusing on frequencies that showed large absolute changes in PSD level across gestation. In addition, *t*-values associated with the regression slope at each frequency were calculated as reference statistics indicating the consistency of the relationship between gestational age and PSD level. These metrics were then used to select gestational age-related frequencies for the multivariate analysis, with frequencies showing the largest absolute β1 values and consistent associations (based on *t*-values) being selected. Specifically, this frequency-selection procedure was performed as a data-driven exploratory screening across the entire analyzed spectrum (30–1000 Hz) to identify individual frequency components with the steepest regression slopes (highest absolute β1 values) during the second trimester. The quantitative screening criteria for feature selection were defined as the frequencies exhibiting the maximum absolute regression slope (|β_1_|) paired with a statistical consistency cutoff of |*t*| ≥ 2.0. This threshold was selected because a |*t*| ≥ 2.0 approximately corresponds to a statistical significance level of α = 0.05 for this sample size, providing a practical baseline to identify prominent spectral features while minimizing type II errors. Because adjacent Fourier frequency components are highly correlated (collinear), conventional strict multiple-comparison corrections, such as the Bonferroni method, were not applied, so as to avoid excessive type II errors. Instead, we selected frequency points that showed both statistical consistency and biological continuity across a broader band (400–600 Hz). By targeting these contiguous spectral trends rather than isolated significant noise spikes, this exploratory approach reduces the risk of capturing stochastic overfitting and increases the likelihood that the selected frequencies reflect stable physiological patterns. To address within-subject correlation from repeated measurements, sensitivity analyses were subsequently conducted on three uncrossed, independent sub-cohorts restricted to initial subject entries.

### 2.4. Multivariate Analysis

The acoustic data were converted to comma-separated values (CSV) format using SpectraPLUS-SC (Pioneer Hill Software LLC, Poulsbo, WA, USA) to ensure compatibility with Vektor Direktor^TM^ (KAX Group, Penrith, Australia). The resulting CSV files were then imported into the software for multivariate analysis, including PCA and PLSR. Prior to multivariate analysis, all variables were standardized to minimize the influence of differences in measurement conditions and inter-individual variability in scale. PCA and PLSR were then performed on the standardized data.

PCA was conducted on a multivariate dataset that included PSD levels at the gestational age-related frequencies together with clinical variables, namely maternal heart rate, gestational age, pre–pregnancy BMI, mean blood pressure, maternal age, hemoglobin concentration closest to the time of recording, and maternal body weight. In addition, PLSR was performed by constructing an explanatory *X*-matrix comprising the clinical parameters and a response *Y*-matrix containing the PSD levels at the gestational age-related frequencies [14,15,16,17,18,19]. PLSR component selection was based on leave-one-out cross-validation (LOO-CV), and the number of latent factors retained was determined by the minimum predicted residual error sum of squares (PRESS). The proportion of variance explained by each latent component (*R*^2^*X* and *R*^2^*Y*), the cross-validated predictive variance (*Q*^2^), and the root-mean-square error (RMSE) are reported.

### 2.5. Ethics Approval

This study was approved by the Ethics Committee of Hokkaido University (approval number: No. 024–0479) on 12 March 2025. Written informed consent was obtained from all participants prior to participation.

## 3. Results

A total of 60 acoustic recordings obtained from 44 participants were included in the analysis. Among the 44 unique participants, 33 contributed a single recording, while 11 underwent multiple recordings (7 participants with 2 recordings, 3 participants with 3 recordings, and 1 participant with 4 recordings), resulting in a total of 60 observations distributed equally across the second (*n* = 30) and third (*n* = 30) trimesters. The clinical characteristics of the study participants and recordings are summarized in Table 1. The mean maternal age was 32.9 ± 5.5 years (range, 19–41 years), and the mean BMI was 21.1 ± 2.0 kg/m^2^. 27 participants (61.4%) were primiparous. No adverse events occurred during acoustic recording (Table 1).

### 3.1. Temporal Changes in TRIAS Within Individual Participants

**Figure 1:**
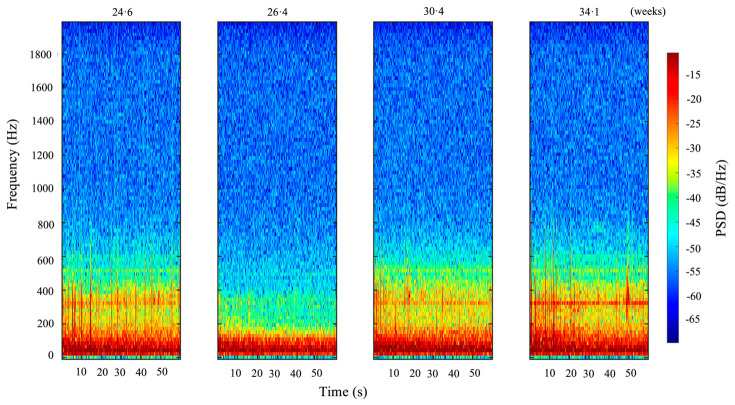
Spectrogram of TRIAS (0–2000 Hz) within a single participant. Approximately 60-second recordings of TRIAS obtained from the same participant at four time points (24 weeks 4 days, 26 weeks 3 days, 30 weeks 3 days, and 34 weeks 1 day of gestation) are displayed using time-frequency analysis. The color bar represents PSD (dB/Hz). PSD levels in the 200–600 Hz range decreased from 24 weeks 4 days to 26 weeks 3 days, showed a transient increase at 30 weeks 3 days, and exhibited a relatively smaller change at 34 weeks 1 day.

To examine temporal changes in TRIAS associated with advancing gestational age, multiple recordings obtained from the same participant were analyzed. PSD levels varied over the course of pregnancy, with noticeable changes observed particularly in the frequency range of approximately 200–600 Hz (Figure 1).

### 3.2. Sensitivity Analysis for Within-Subject Correlation

Before conducting the primary group-level and multivariate analyses on the full dataset (*n* = 60), we evaluated the statistical robustness of our framework against potential within-subject correlation caused by repeated measurement. Three distinct sensitivity analyses were performed on independent sub-cohorts restricted to initial subject entries. First, an analysis of the initial baseline recording from each unique individual across the entire study (*N* = 44) demonstrated that, while the 700–900 Hz band reached statistical significance (*p* < 0.05), the absolute magnitude of the regression slope (β_1_) remained near zero, indicating a negligible physiological effect and representing a practical plateau in this frequency range (Figure S2a). Second, the independent second trimester cohort (*N* = 26), using only the first recording within this trimester, confirmed the negative slope at 478.5 Hz and 521.5 Hz (*p* < 0.05; Figure S2b). Third, a cohort of unique individuals who entered the protocol strictly in the third trimester (*N* = 18) showed that the 400–600 Hz band remained stable (*p* > 0.05; Figure S2c). These comparative contrasts suggest that the mid-frequency attenuation represents a physiological characteristic specific to the second trimester rather than a result of repeated measures confounding. Therefore, to maximize statistical power and capture fine-grained temporal dynamics across groups, all subsequent primary frequency-domain evaluations, OLS regressions, and multivariate modeling (PCA and PLSR) were conducted using the full 60-recording dataset.

### 3.3. Frequency-Domain Analysis and Identification of Major Spectral Bands

**Figure 2:**
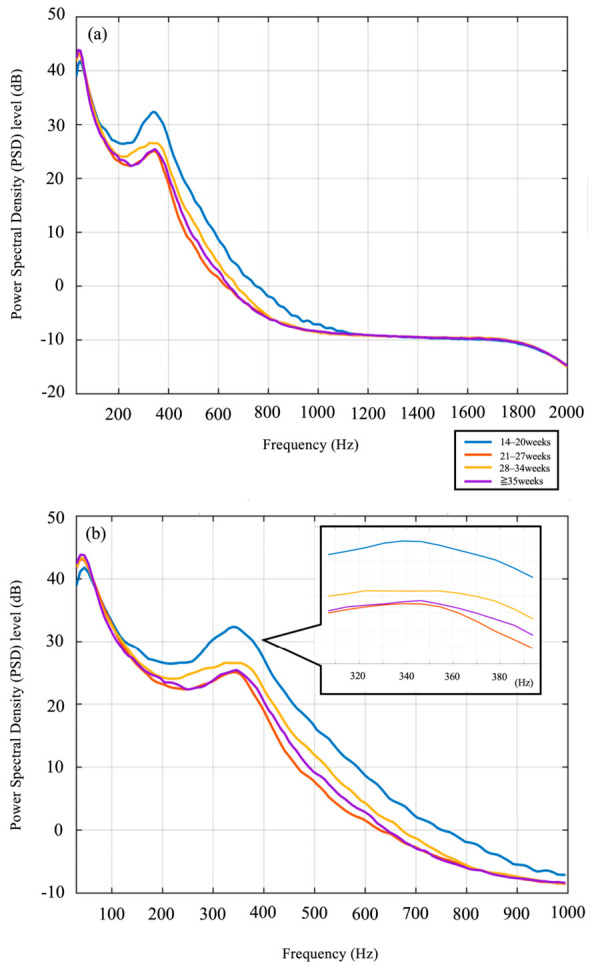
Changes in the mean power spectrum across gestation. (**a**) A total of 60 recordings obtained from 44 participants were analyzed. Recordings obtained between 14 and 38 weeks of gestation were categorized into four gestational age groups at 7-week intervals: 14–20 weeks (blue; *n* = 10 recordings from *N* = 10 participants), 21–27 weeks (orange; *n* = 20 recordings from *N* = 16 participants), 28–34 weeks (yellow; *n* = 18 recordings from *N* = 14 participants), ≥35 weeks (purple; *n* = 12 recordings from *N* = 11 participants). After normalization so that the mean PSD across the 30–2000 Hz band was set to 0 dB for each recording, the mean PSD curve for each group was calculated. (**b**) Because little variation was observed above 1000 Hz, the spectrum was further expanded for the 30–1000 Hz range. Differences around 300–400 Hz became more evident; although the peak location was similar across groups, the PSD level in the 300–400 Hz range changed with advancing gestational age.

To visually assess how the spectral characteristics of TRIAS change over the course of pregnancy, PSD levels were calculated across the full frequency range of 30–2000 Hz. Recordings were grouped into four categories according to gestational age using 7-week intervals, and the mean PSD spectrum was calculated for each group (Figure 2a). Across all gestational age groups, a consistent spectral peak was observed at approximately 300–400 Hz. In contrast, frequency components above 1000 Hz showed little variation with gestational age. Therefore, subsequent analyses were restricted to the 30–1000 Hz frequency range (Figure 2b).

### 3.4. Analysis of Temporal Changes in PSD Levels

**Figure 3:**
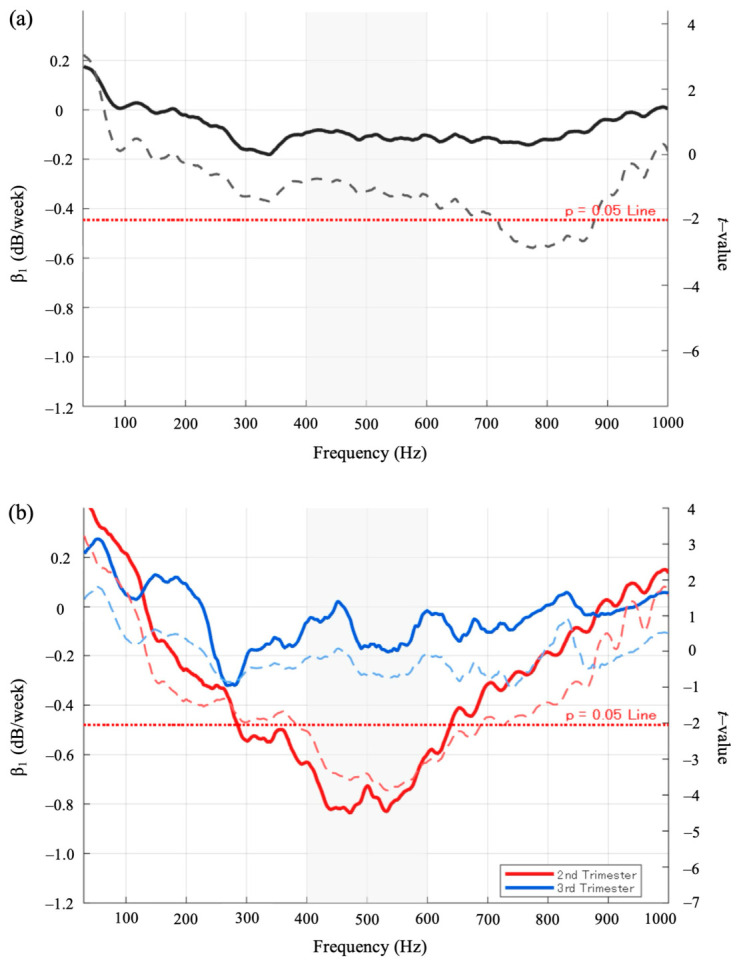
Frequency-specific associations between PSD levels and gestational age. (**a**) Temporal rate of change in PSD level (β_1_, dB/week; solid line) and corresponding statistical values (*t*-values; dashed line) across the entire cohort (14–38 weeks) estimated via OLS regression (*N* = 44 participants, total recordings = 60). A pronounced negative slope was observed in the 300–400 Hz range, particularly near 332.0 Hz. (**b**) Trimester-specific OLS regression analysis for the second trimester (red line; *N* = 26 participants, 30 recordings) and third trimester (blue line; *N* = 21 participants, 30 recordings). During the second trimester, consistently negative slopes were observed in the 400–600 Hz range, with particularly prominent changes near 478.5 Hz and 521.5 Hz. In contrast, slopes during the third trimester were generally distributed around 0. In both panels, the horizontal red dashed line marks the alpha = 0.05 significance threshold (|*t*| ≥ 2.0), and the gray shaded areas highlight the prominent 400–600 Hz mid-frequency band showing these gestational alterations.

The association between gestational age and PSD level was evaluated for each frequency. For each frequency, OLS regression analysis was performed, in which the regression slope represented the rate of change in PSD level with advancing gestational age (β_1_, dB/week). The corresponding *t*-values were also calculated and reported as an index of the consistency of the relationship between gestational age and PSD level. As a result, a pronounced negative slope was observed in the 300–400 Hz frequency band, with the strongest negative association identified near 332.0 Hz (*t* = −1.6, 95% confidence interval [CI]: −0.46 to 0.06, *p* = 0.12) (Figure 3a).

### 3.5. Comparison Between the Second and Third Trimesters

We compared the acoustic changes between the second and third trimesters. OLS regression analysis revealed that, during the second trimester, consistently negative regression slopes were observed in the 400–600 Hz frequency band. In particular, the strongest negative associations were observed near 478.5 Hz (*t* = −3.7, 95% confidence interval [CI]: −1.37 to −0.39, *p* < 0.05), and 521.5 Hz (*t* = −4.1, 95% confidence interval [CI]: −1.35 to −0.45, *p* < 0.05) (Figure 3b). In contrast, during the third trimester, the regression slopes were clustered around 0 dB/week across this frequency range, indicating little to no change in PSD level with advancing gestational age (Figure 3b). These results indicate that PSD levels in the 400–600 Hz range decrease predominantly during the second trimester, whereas this trend attenuates in late pregnancy. To further verify that these findings reflect a contiguous spectral trend rather than isolated stochastic noise spikes, a supplementary regression analysis was conducted using the averaged band-level PSD within the broader 400–600 Hz range restricted to the independent second trimester cohort (*N* = 26). This band-level feature confirmed a statistically highly significant decline during the second trimester (β_1_ = −0.7007 dB/week, *t* = −3.4660, *p* < 0.05, 95% CI: −1.1180 to −0.2835), demonstrating a continuous shift within this mid-frequency range.

### 3.6. Principal Component Analysis (PCA)

**Figure 4:**
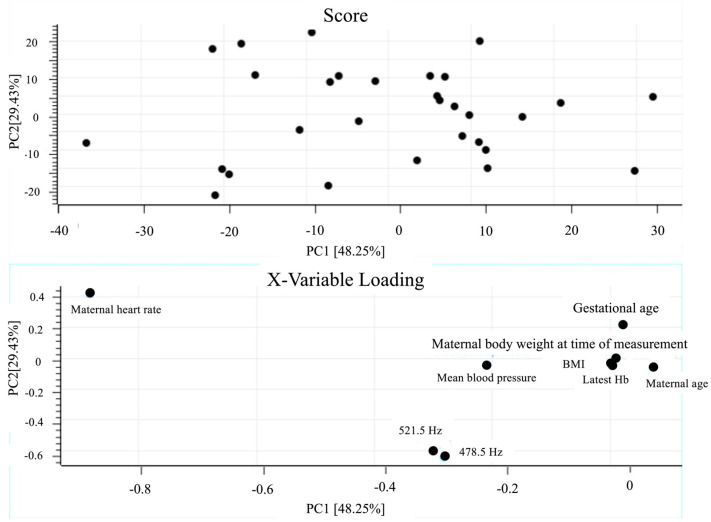
Principal component analysis (PCA) during the second trimester (analyzed data restricted to the second trimester recordings, *N* = 26 participants, 30 total recordings). PCA was performed on data obtained during the second trimester, using a multivariate dataset that included clinical variables (e.g., maternal heart rate and gestational age) together with PSD levels at gestational age-related frequencies (478.5 Hz and 521.5 Hz) identified by OLS analysis. In the loading plot, maternal heart rate showed the largest contribution to PC1 (=−0.88). This analysis enabled acoustic levels at 478.5 Hz and 521.5 Hz and clinical variables to be represented within the same multivariate space.

PCA was performed on a multivariate dataset that included PSD levels at 478.5 Hz and 521.5 Hz—frequencies that showed marked changes during the second trimester—together with clinical variables. In the loading plot, maternal heart rate showed the largest contribution to PC1 (=−0.88). PC1, PC2, and PC3 explained 48.25%, 29.43%, and 12.51% of the total variance, respectively, with a cumulative explained variance of 90.19%. PSD levels at 478.5 Hz and 521.5 Hz also showed moderately large negative loadings on PC1 (≈−0.3), indicating that these acoustic components varied in the same direction as maternal heart rate (Figure 4).

### 3.7. Partial Least Squares Regression (PLSR)

**Figure 5:**
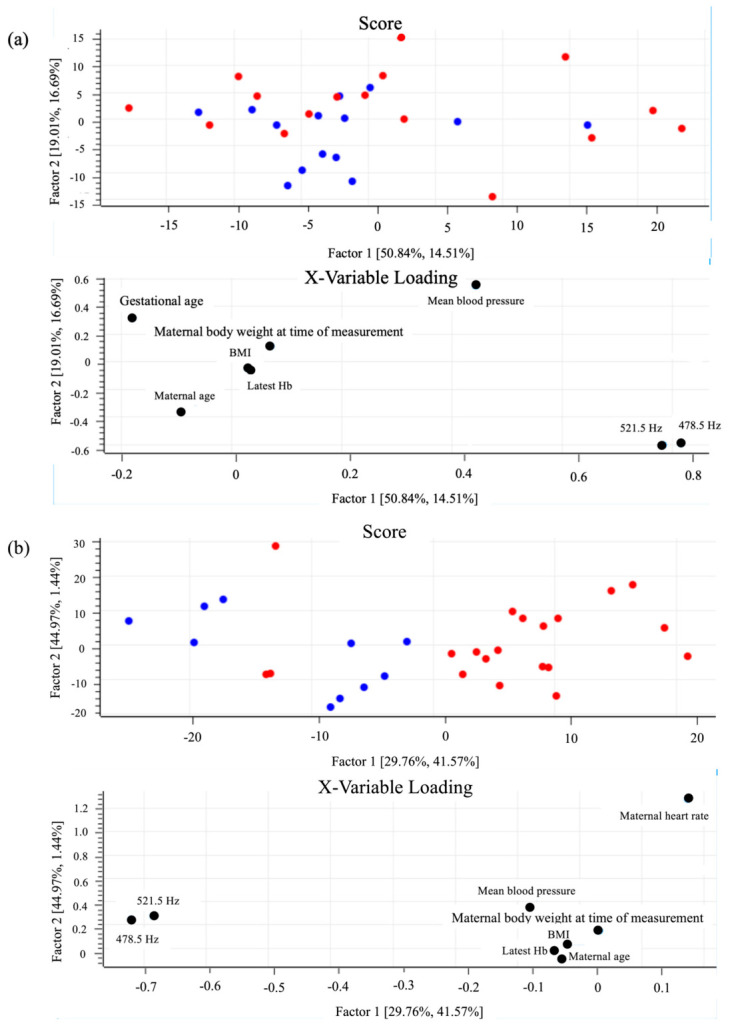
Decoding pregnancy progression and maternal heart rate via partial least squares regression (PLSR) during the second trimester (analyzed data restricted to the second trimester recordings, *N* = 26 participants, 30 total recordings; evaluated using leave-one-out cross-validation). (**a**) PLSR using maternal heart rate as the response variable. While acoustic components (478.5 Hz and 521.5 Hz) are located in the positive direction of Factor 1 in the loading plot, the score plot shows an overlapping distribution between the low (blue) and high (red) maternal heart rate groups. (**b**) PLSR using gestational age as the response variable. The two frequency components are positioned in the negative direction of Factor 1, and the score plot demonstrates a clear separation between earlier (blue) and later (red) gestational age groups.

Maternal heart rate represents an immediate physiological indicator of maternal circulatory dynamics, whereas gestational age reflects the temporal progression of fetal and placental maturation. In this study, PLSR was performed using each of these variables as the response variable to examine how acoustic features were associated with distinct physiological aspects. To facilitate visual interpretation of the score plots, maternal heart rate and gestational age were dichotomized at their median values and displayed using different colors. When maternal heart rate was used as the response variable, both frequency components (478.5 Hz and 521.5 Hz) were located in the positive direction of Factor 1 in the loading plot. However, the corresponding score plot did not show a distinct distribution pattern separating the low and high maternal heart rate groups (Figure 5a). In contrast, when gestational age was used as the response variable, the same two frequency components (478.5 Hz and 521.5 Hz) were positioned in the negative direction of Factor 1 in the loading plot. In the corresponding score plot, recordings from an earlier gestational age were predominantly distributed on the negative side of Factor 1. This distribution was consistent with the frequency-specific OLS regression analyses that identified gestational age-related frequencies (Figure 3a,b and Figure 5b). The PLSR model for gestational age retained 2 latent variables selected via leave-one-out cross-validation, explaining 67.8% of the *Y*-variance with a cross-validated predictive variance (Q^2^) of 0.584, and a root-mean-square error (RMSE) of 1.942 weeks.

## 4. Discussion

The main findings of this study can be summarized in four points. First, the mean spectrum of TRIAS consistently exhibited a common peak around 300–400 Hz regardless of gestational age. Second, frequency-specific OLS analysis demonstrated a marked decline in PSD levels around 478.5 Hz and 521.5 Hz during the second trimester, whereas these changes were minimal during the third trimester. Third, PCA incorporating PSD levels at these frequencies together with clinical variables revealed that maternal heart rate exhibited the largest negative contribution to PC1, with the gestational age-related acoustic components varying in the same direction. Fourth, PLSR indicated that these acoustic components could potentially decode pregnancy progression with a predictive accuracy of 1.942 weeks, while showing an overlapping distribution when modeled against maternal heart rate, thereby highlighting the gestational age-specific nature of the observed acoustic alterations.

### 4.1. Acoustic Changes Associated with Gestational Age

In this study, to quantitatively evaluate the relationship between acoustic components contained in TRIAS and gestational age, the linear association between PSD levels at each frequency and gestational age was assessed using OLS regression analysis, and characteristic frequencies most strongly associated with gestational progression were identified.

Spectrogram-based visualization of temporal changes demonstrated that acoustic variations across gestation did not follow a simple monotonic trend but rather showed fluctuations with a certain degree of periodicity. For example, in one participant, PSD levels in the 200–600 Hz range decreased from 24 weeks and 4 days to 26 weeks and 3 days of gestation, increased again at 30 weeks and 3 days, and subsequently exhibited minimal change at 34 weeks and 1 day (Figure 1). These observations suggest that TRIAS do not change in a purely linear manner but may instead exhibit repeated increases and decreases as gestational advances. Physiologically, these non-monotonic variations could potentially be associated with the dynamic, non-linear remodeling of the intrauterine environment—such as the non-uniform expansion of the amniotic fluid volume and fluctuations in uterine wall tension. Recent high-resolution in vivo transmission models across the human audio range have demonstrated that intrauterine tissues and fluid pockets function as a complex, fine-frequency acoustic filter [20]. As detailed later in this section, these macroenvironmental structural shifts and the geometric positioning of the stethoscope relative to the maternal vasculature collectively alter transmission loss and resonance characteristics over gestation, resulting in the observed wave-like patterns rather than a simple monotonic decline.

Therefore, rather than relying solely on visual inspection, quantifying the relationship between individual frequency components and gestational age using OLS regression, identifying gestational age-related frequencies, and subsequently incorporating them into multivariate analyses was considered useful for defining these acoustic signatures with the present dataset.

### 4.2. Extraction of Gestational Age-Related Frequencies and the Significance of Multivariate Analysis

PCA was performed using the gestational age-related frequencies selected by OLS analysis together with clinical variables. In the second trimester, maternal heart rate showed the strongest contribution to the principal component that explained the overall structure of the dataset. The significance of this finding lies in visualizing the possibility that the overall variability of sounds recorded from the abdominal wall may reflect the maternal cardiac rhythm.

Furthermore, in the PLSR analyses, regardless of whether maternal heart rate or gestational age was used as the response variable, the PSD levels at 478.5 Hz and 521.5 Hz were clearly positioned as covarying components in the loading plots, albeit oriented in opposite directions along Factor 1 (positive for maternal heart rate and negative for gestational age). When maternal heart rate was used as the response variable, no distinct group separation was observed in the corresponding score plot. In contrast, when gestational age was used as the response variable, the recordings from earlier gestational age were predominantly distributed on the negative side of Factor 1, demonstrating a clear separation from the later gestational age groups. These findings suggest that specific frequency components contained in TRIAS may be associated with both maternal circulatory indicators and the progression of pregnancy, representing different physiological dimensions.

An important aspect of this study is that the relationship between acoustic characteristics at gestational age-related frequencies and clinical indicators was explored by combining frequency selection based on OLS analysis with multivariate analyses using PCA and PLSR. This approach enabled visualization of patterns in which multiple frequency components and clinical variables change simultaneously—patterns that are difficult to capture using univariate analyses alone. This analytical framework suggests that TRIAS may serve as an indicator encompassing multiple physiological signals and may provide a basis for future evaluation of pregnancy progression and the development of non-invasive maternal–fetal monitoring methods [14,16,18,19].

### 4.3. Physiological Background of the Decrease in PSD Levels Around 400–600 Hz During the Second Trimester

The acoustic changes observed in this study were primarily concentrated in the frequency range of approximately 400–600 Hz, with a sustained decrease in PSD levels in this band particularly evident during the second trimester. This finding suggests that acoustic components around 400–600 Hz may be relatively attenuated as pregnancy progresses in TRIAS. This tendency is consistent with previous studies reporting a predominance of lower-frequency components in transabdominally recorded intrauterine acoustic signals [6,7,8].

In the present study, acoustic recordings were obtained from the lower maternal abdomen, and macro-environmental alterations associated with advancing gestation may influence the propagation characteristics of acoustic signals detected at the abdominal wall. Acoustic transmission through these structural changes may produce frequency-dependent attenuation, whereby relatively preserved frequency components appear as characteristic features of the acoustic spectrum observed at the abdominal surface. Indeed, certain frequency bands in the present study exhibited minimal change with advancing gestational age, suggesting that these components may be relatively less affected by these structural shifts and therefore may provide limited information as indicators of gestational progression. In contrast, frequency components that showed gestational variation are likely to reflect the influence of these physiological changes and may be regarded as characteristic acoustic features of TRIAS.

The biological and physical basis for the selective attenuation during the second trimester within the 400–600 Hz range (specifically at 478.5 Hz and 521.5 Hz) can be interpreted physiologically as follows. During the second trimester, rapid uterine hypertrophy combined with an exponential increase in amniotic fluid volume alters the intrauterine acoustic transmission medium [5,8,20]. Physiologically, sonographic tracking of normal singleton gestations demonstrates that the amniotic fluid index progressively expands starting from 14 to 16 weeks’ gestation and continuously increases throughout the second trimester to reach its peak at approximately 26–28 weeks [21]. Chronologically, this substantial accumulation of fluid expands the gestational cavity, which physically functions as an enhanced acoustic low-pass filter. This bio-acoustic boundary preferentially scatters and absorbs higher mid-frequency bands (400–600 Hz) while leaving lower structural murmurs (<200 Hz) relatively unattenuated [6,7,20]. This masking mechanism is directly supported by high-fidelity in vivo models demonstrating that fluid-filled gestational structures introduce significant frequency-specific insertion loss across the audio spectrum as the geometry shifts [20]. Furthermore, the structural maturation of the placenta and the physical expansion of the maternal abdominal wall increase the mechanical damping coefficient of the tissue interface [5,6]. Alternatively, this attenuation may reflect a relative reduction in broad-spectrum maternal vascular turbulence or a structural shielding effect as the growing uterus alters the geometric positioning of the digital stethoscope relative to the major retroperitoneal maternal vessels [9,10]. Consequently, these specific frequencies could be considered exploratory statistical markers of structural changes within the gestational cavity [16].

Furthermore, PLSR analysis indicated a covariance structure between PSD levels at 478.5 Hz and 521.5 Hz and maternal heart rate. This finding suggests that acoustic components originating from the maternal cardiac cycle may be transmitted and attenuated through the abdominal wall and intrauterine milieu, thereby contributing to acoustic signals observed in the 400–600 Hz frequency range. Collectively, these observations support the notion that TRIAS represent a composite acoustic signal composed of multiple overlapping physiological sound sources, including maternal circulation, respiration, and intestinal activity, rather than signals derived from a single origin.

### 4.4. Clinical Implications and Future Perspectives of Multivariate Analysis

In this study, chemometrics-based multivariate analytical methods were applied to abdominal acoustic data in the field of obstetrics. Although chemometrics has been widely used in medical research for metabolomic analysis and statistical evaluation of various biosignals, to our knowledge, no previous reports have integratively analyzed the relationships between TRIAS, gestational age, and maternal circulatory indicators. By integrating the multicomponent acoustic characteristics of TRIAS with clinical variables and describing their relationships within a multivariate analytical framework, the present study demonstrates a potential new application of chemometrics in perinatal research. The introduction of multivariate analysis offers several advantages. First, it enables the characterization of TRIAS, in which multiple frequency components change simultaneously, in a manner that cannot be adequately captured using single-frequency or univariate analysis [16,22]. Second, PCA and PLSR provide strong interpretability in terms of visualization and regression structure and are less “black-box” than many machine-learning approaches, which may facilitate their acceptance in clinical practice [14,23]. Third, multivariate models may help account for variability arising from measurement conditions and inter-individual differences, offering a foundation for future decentralized monitoring applications [24,25,26].

The findings of the present study suggest that this framework may complement existing evaluation methods that rely primarily on single indicators, such as fetal heart rate monitoring and Doppler assessment, by enabling an integrated interpretation of broadband acoustic signatures for enhanced maternal–fetal monitoring. In particular, multivariate characterization of frequency-specific features—such as the reduction in PSD levels in the 400–600 Hz range during the second trimester and the relatively smaller changes observed in late pregnancy—may allow a more comprehensive assessment of physiological changes associated with gestational progression. Importantly, the present study represents an exploratory feasibility evaluation rather than a clinically validated diagnostic platform. While these signatures show robust statistical association, further large-scale, prospective validation in unselected cohorts—incorporating direct comparisons with established obstetric metrics and medical outcomes—is essential before any clinical translation or home-monitoring device integration can be realized. Future studies must explicitly separate demonstrated feasibility from potential future applications by evaluating device-to-device reproducibility and performance in high-risk pregnancies.

Future studies are required to evaluate the generalizability of the proposed model using independent cohorts and datasets that include pregnancies with a wider range of clinical conditions. The internal stability and predictive performance of our PLSR model were evaluated using a leave-one-out cross-validation approach within the present dataset. Subsequent external validation using independent, larger prospective cohorts will be needed to establish the stability, generalizability, and reproducibility of these acoustic metrics. Taken together, the present analytical framework, which combines the extraction of gestational age-related frequencies with multivariate integration of clinical indicators, may provide a foundation for the development of non-invasive monitoring approaches in perinatal medicine.

### 4.5. Methodological Limitations and Inferred Physiological Mechanisms

This study has several limitations.

First, because recordings were obtained during routine clinical practice, ambient noise could not be completely eliminated despite the use of standardized protocols.

Second, we excluded participants with a pre-pregnancy BMI ≥ 25 kg/m^2^, hypertensive disorders of pregnancy, or gestational diabetes mellitus to isolate baseline acoustic signatures strictly within a low-risk population. While these metabolic and vascular factors alter hemodynamics and abdominal wall acoustics, this restriction limits the direct generalizability of our current spectral signatures to an unselected obstetric population. However, establishing this normative template is a prerequisite for subsequent comparative studies in high-risk cohorts to evaluate the utility of the framework for clinical screening.

Third, the current design cannot definitively attribute the signal to a specific biological source. Because TRIAS are a composite of concurrent maternal (cardiovascular, respiratory, intestinal) and fetal-placental signals, the anatomical origin of the second-trimester attenuation at 478.5 and 521.5 Hz remains uncertain. Without simultaneous reference signals—maternal electrocardiography (ECG), continuous cardiotocography, or Doppler flow metrics—we cannot determine whether these shifts reflect changes in fetal physiology or structural changes in the acoustic transmission path, such as uterine hypertrophy, amniotic fluid dynamics, or placental vascular remodeling. These frequency components should therefore be interpreted as gestational-age-associated spectral signatures rather than validated biomarkers of fetal well-being. Specifically, the lack of synchronized fetal heart rate (FHR) tracking prevents us from isolating localized fetal acoustic events from the dominant maternal cardiorespiratory background. If concurrent FHR or quantitative fetal activity metrics had been acquired, these fetal-specific parameters could have been integrated into PCA and PLSR modeling in the same manner as maternal parameters. This approach would potentially allow us to evaluate the specific spectral coefficients corresponding to fetal cardiac dynamics and mathematically separate them from maternal circulatory contributions. Resolving this signal-separation bottleneck is an essential prerequisite for translating the TRIAS framework from an indirect indicator of gestational progression into a high-fidelity platform for direct, non-invasive fetal monitoring. In addition, although our primary analysis treated recordings as independent, repeated measurements from a subset of participants introduce potential within-subject correlation. Validation in larger, longitudinal, unselected cohorts, including high-risk pregnancies, will therefore be needed to confirm the robustness of this framework. Although our sensitivity analyses on independent sub-cohorts reduced the influence of these repeated measurements, they do not formally replace a mixed-effects modeling approach; the latter should be applied in future studies with a more balanced longitudinal design.

Fourth, our spectral normalization method relied on subtracting the mean broadband PSD for each recording. While this approach effectively eliminates inter-individual variances in absolute sound intensity caused by operator pressure or tissue thickness, it inherently targets relative spectral shapes rather than absolute power variations. Alternative methods, such as participant-specific *z*-score normalization, could scale variance uniformly but risk distorting the physical covariance structure among adjacent frequencies. The potential loss of absolute broadband gain information remains a minor limitation of the current signal processing pipeline.

Furthermore, achieving the transition from controlled laboratory recordings to real-world clinical or remote monitoring environments necessitates ensuring acoustic robustness against diverse environmental artifacts. Optimizing the acoustic coupling and sensor positioning for non-expert users, along with navigating standard medical device evaluation protocols, represents the logical next phase for integrating the TRIAS framework into routine prenatal care.

## 5. Conclusions

In this study, by combining frequency–domain analysis with multivariate approaches (PCA and PLSR), we quantitatively characterized the acoustic signatures associated with gestational progression in transabdominally recorded intrauterine acoustic signals obtained using a digital stethoscope. In particular, the pronounced reduction in PSD levels in the 400–600 Hz frequency range (specifically at 478.5 Hz and 521.5 Hz) observed during the second trimester, together with the identification of maternal heart rate as a major contributor in the PCA, visualized distinct physiological processes as opposing multivariate vectors. This framework captures overlapping maternal-fetal signals that are difficult to separate using conventional univariate analyses, suggesting the potential of chemometrics in perinatal medicine.

The present results suggest that TRIAS may serve as a useful indicator reflecting multiple physiological processes of both the mother and fetus and support its potential role as a complementary assessment approach to existing monitoring methods. Future studies are needed to enhance the generalizability and reproducibility of this approach through validation in datasets that include complicated pregnancies, standardization of recording procedures, and expansion of sample size. Furthermore, development of simple and consistent analytical algorithms will be important to facilitate application in routine clinical practice and perinatal remote monitoring.

The analytical framework presented in this study may provide a foundation for a novel perinatal monitoring approach that enables a more integrated assessment of maternal and fetal conditions and holds promise for future clinical implementation.

## Figures and Tables

**Table 1:** Clinical Characteristics.

Characteristic	Total	2nd Trimester	3rd Trimester
Total recordings, *n*	60	30	30
Total participants, *N*	44	26	21
Maternal age (years)	32.9 (5.5)	31.8 (5.2)	34.0 (5.8)
Pre–pregnancy BMI (kg/m^2^)	21.1 (2.0)	20.9 (1.9)	21.3 (2.1)
Primiparous, *N* (%) *	27 (61.4%)	11 (42.3%)	16 (76.2%)
Gestational age (weeks)	26.4 (6.8)	19.2 (2.4)	33.6 (2.5)
Maternal heart rate (bpm)	84.3 (9.3)	83.1 (9.6)	85.5 (9.0)
Mean blood pressure (mmHg)	80.5 (7.7)	78.8 (7.8)	82.2 (7.2)
Hemoglobin (g/dL)	11.6 (0.8)	12.0 (0.8)	11.2 (0.7)

Data are mean (SD) or *n* (%). SD: standard deviation. * Calculated based on the number of unique participants (*N* = 44). Other variables are based on the total number of recordings (*n* = 60). Because some participants were recorded during both the second and third trimesters, the sum of the trimester-specific participant counts (26 + 21) exceeds the total number of unique participants (*N* = 44).

## Data Availability

The datasets generated and/or analyzed during the current study are not publicly available due to the sensitive nature of the data and to protect participant privacy. However, anonymized data may be made available from the corresponding author upon reasonable request. The data are securely stored in the Department of Obstetrics, Hokkaido University Hospital.

## Supplementary Materials

The following supporting information can be downloaded at: https://www.mdpi.com/article/10.3390/s26134150/s1, Figure S1: Comprehensive 2000 Hz spectral scans for sensitivity analyses restricted to independent initial recordings; Figure S2: Detailed 1000 Hz expanded profiles of the trimester-specific sensitivity analyses.

## Abbreviations

The following abbreviations are used in this manuscript:

TRIAS	Transabdominally recorded intrauterine acoustic signals
PSD	Power spectral density
OLS	Ordinary least squares
PCA	Principal component analysis
PLSR	Partial least squares regression
