# Supplementary material for: Multivariate Spectral Analysis of Transabdominally Recorded Intrauterine Acoustic Signals (TRIAS) Across Gestation
Source: Sensors (Basel). 2026 Jul 1;26(13):4150. doi: 10.3390/s26134150 (PMC13363705; doi:10.3390/s26134150)
Supplement: Supplementary file 1 [file sensors-26-04150-s001.zip › sensors-4346086-supplementary.pdf]

Supplementary Materials

Article

Multivariate Spectral Analysis of Transabdominally Recorded Intrauterine Acoustic Signals (TRIAS) Across Gestation

Ryo Tamaki, Fuyuka Igarashi, Ryutaro Yamamoto, Hiroshi Asano, Akira Oku, Keiichiroh Akabane, Kiwamu Noshiro, Ami Hosokawa, Yoshihiro Saito, Hidemichi Watari and Takeshi Umazume *

Department of Obstetrics, Hokkaido University Hospital, Kita–ku N15 W7, Sapporo 060-8638, Japan

* Correspondence: takeuma@med.hokudai.ac.jp; Tel.: +81-11-706-5941;
Fax: +81-11-706-7711

This document provides supplementary research data and sensitivity analysis results to support the primary findings of the main manuscript.

Figure S1

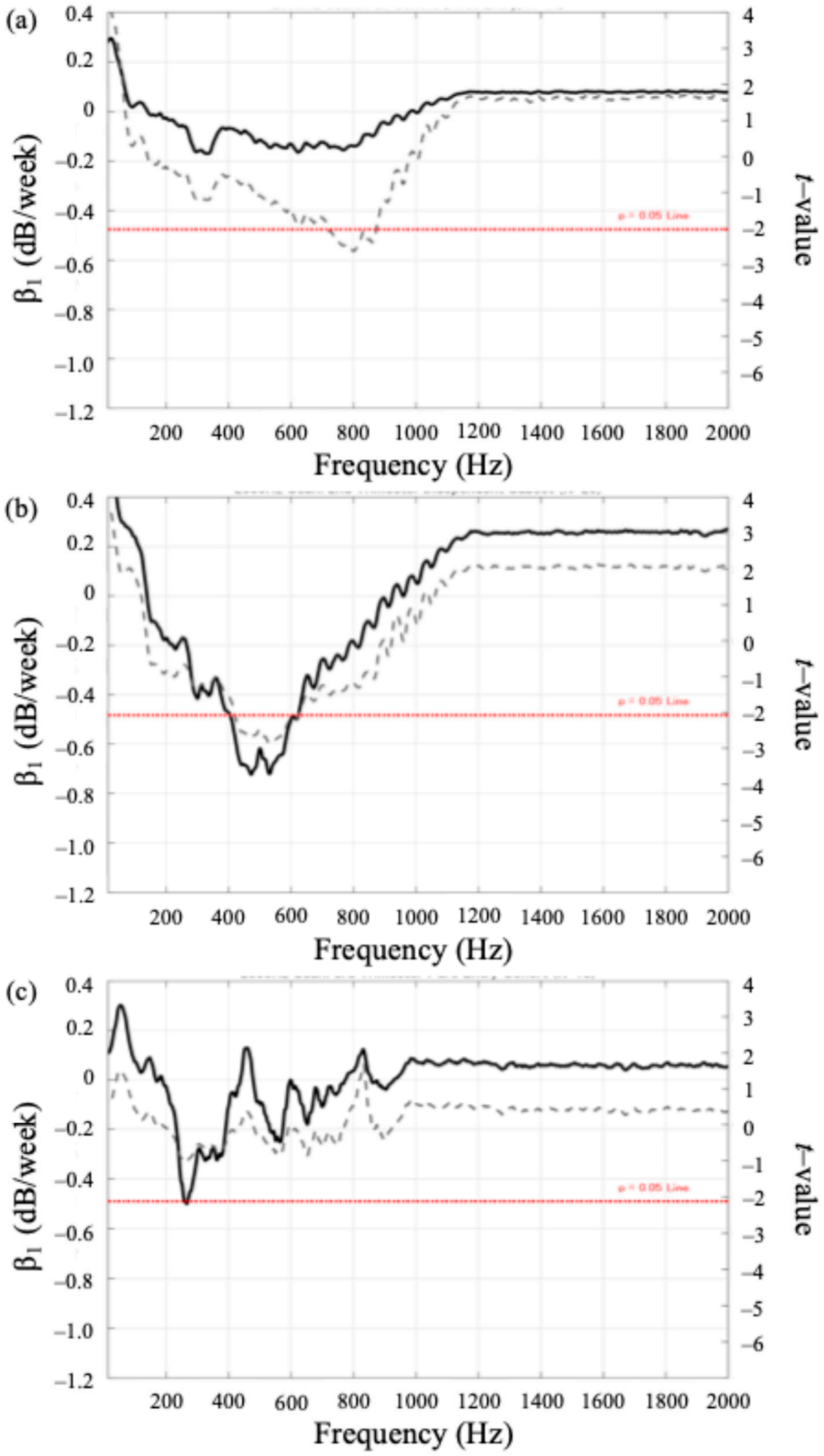

(a)
(b)
(c)
β1 (dB/week)
t–value
Frequency (Hz)
0.4
0.2
0
−0.2
−0.4
−0.6
−0.8
−1.0
−1.2
4
3
2
1
0
−1
−2
−3
−4
−5
−6
200
400
600
800
1000
1200
1400
1600
1800
2000

Figure S1. Comprehensive 2000 Hz spectral scans for sensitivity analyses restricted to independent initial recordings.

To rigorously evaluate the potential confounding effect of within–subject correlation from repeated longitudinal measurements, sensitivity analyses were performed by restricting the dataset to a single, independently selected initial recording from each unique participant across different cohort subsets, scanned across the full 2000 Hz spectrum. The temporal rate of change in PSD level (β_1, dB/week; solid line) and the corresponding statistical consistency (*t* –values; dashed line) are plotted against frequency.

(a) Full Cohort First Entry (*N* = 44): OLS regression across the entire 2000 Hz spectrum demonstrates that while the 700–900 Hz band formally crossed the statistical significance threshold ($t \leq -2.0$, $p < 0.05$), the absolute magnitude of the regression slope remained minimal. Above 1000 Hz, the spectrum exhibits a completely flat, non-dynamic trajectory, confirming it represents the electronic/hardware noise floor of the acoustic sensor.

(b) 2nd Trimester Independent Subset (*N* = 26): Restricting the data strictly to the first baseline recording obtained within the second trimester shows that the spectrum above 1000 Hz remains flat and inactive, stable around the noise floor, while the mid-frequency alterations are highlighted below this range.

(c) 3rd Trimester Pure Entry Cohort (*N* = 18): Analyzing unique individuals who entered the protocol strictly in the third trimester confirms a flat noise floor profile above 1000 Hz, with no significant spectral dynamics across the entire high-frequency spectrum.

Figure S2

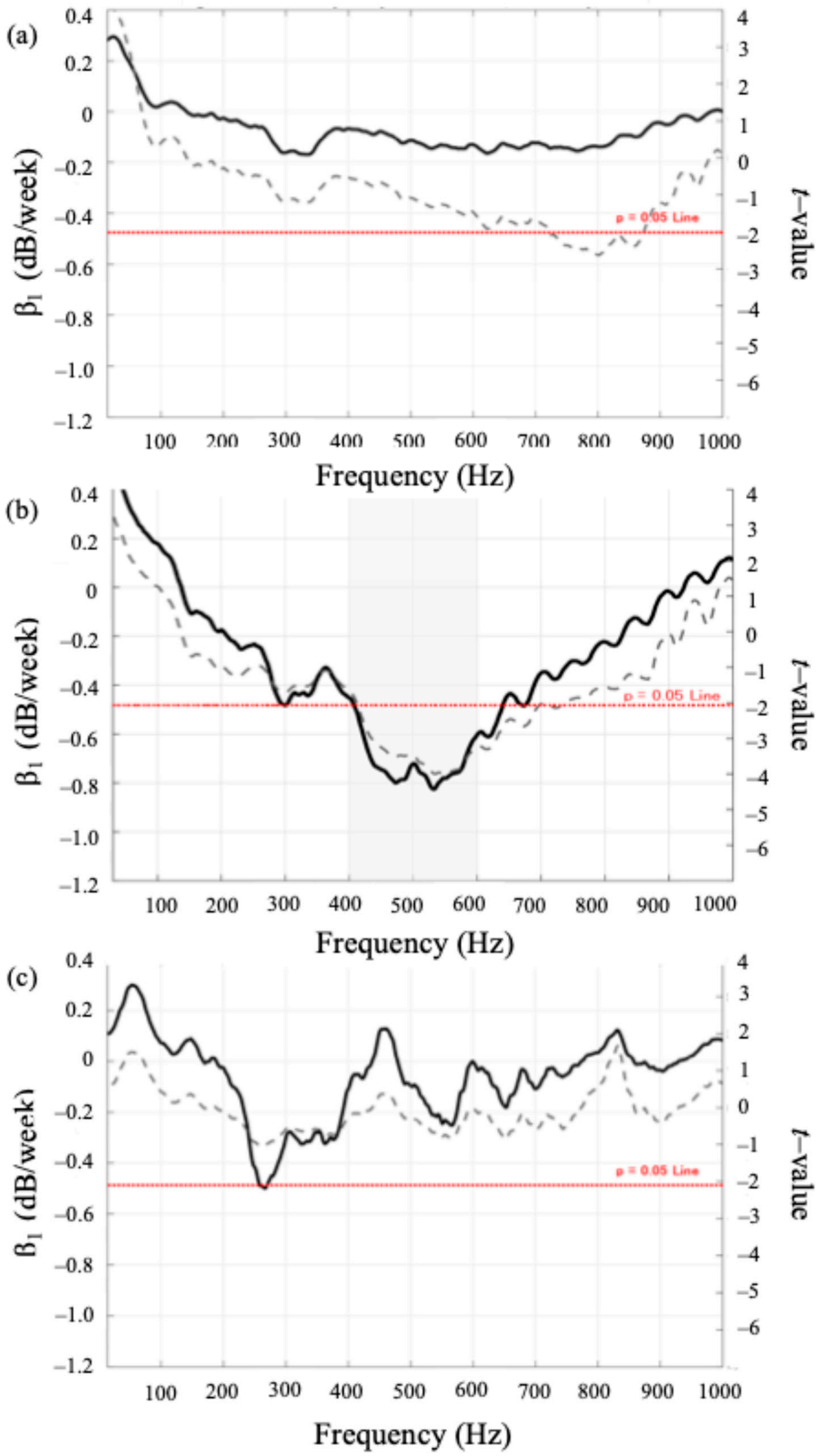
(a)
(b)
(c)
β1 (dB/week)
t–value
Frequency (Hz)
p = 0.05 Line
0.4
0.2
0
–0.2
–0.4
–0.6
–0.8
–1.0
–1.2
4
3
2
1
–1
–2
–3
–4
–5
–6
100
200
300
400
500
600
700
800
900
1000

Figure S2. Detailed 1000 Hz expanded profiles of the trimester-specific sensitivity analyses. To provide high-resolution visualization of the primary physiological changes, the sensitivity analysis results restricted to independent initial recordings (presented in Figure S1) are displayed here focusing on the 100–1000 Hz frequency range, excluding the inactive high-frequency noise floor above 1000 Hz.

(a) Full Cohort First Entry (*N* = 44): The expanded view highlights that although the 700–900 Hz range reaches statistical significance ($t \leq -2.0$), the β_1 slope remains near zero, indicating a practical plateau with minimal biological shift.

(b) 2nd Trimester Independent Subset (*N* = 26): This high-resolution panel clearly demonstrates that the significant PSD decline within the 400–600 Hz range—specifically centered around 478.5 Hz and 521.5 Hz—remains statistically highly consistent ($t < -2.0$, $p < 0.05$) and structurally robust against repeated-measurement bias.

(c) 3rd Trimester Pure Entry Cohort (*N* = 18): The expanded scale securely confirms that the 400–600 Hz frequency band remains entirely stable around 0 dB/week ($p > 0.05$) in the third trimester, showing no residual downward trend.
